# Housing typologies and asthma: a scoping review

**DOI:** 10.1186/s12889-023-16594-8

**Published:** 2023-09-11

**Authors:** Amber Howard, Adelle Mansour, Georgia Warren-Myers, Christopher Jensen, Rebecca Bentley

**Affiliations:** 1 Melbourne School of Population and Global Health, University of Melbourne, Victoria, Australia; 2 Melbourne School of Design, University of Melbourne, Victoria, Australia

**Keywords:** Asthma, Housing, Dwelling conditions, Disparities, Home environments, Risk factors, Respiratory health

## Abstract

Asthma is related to triggers within the home. Although it is recognised that triggers likely occur due to characteristics of housing, these characteristics have not been comprehensively reviewed, and there is a paucity of housing-focused interventions to reduce asthma and asthma symptoms. Following five steps identified by Arksey and O’Malley, we conducted a scoping review of published evidence on the associations between asthma and housing characteristics. We searched three electronic databases (PubMed, Scopus, Web of Science), identifying 33 studies that met our inclusion criteria. Through an iterative approach, we identified nine housing characteristics relevant to asthma onset or exacerbation, categorised as relating to the surrounding environment (location), the house itself (dwelling), or to conditions inside the home (occupancy). We conceptualise these three levels through a housing typologies framework. This facilitates the mapping of housing characteristics, and visualises how they can cluster and overlap to exacerbate asthma or asthma symptoms. Of the three levels in our framework, associations between asthma and locational features were evidenced most clearly in the literature reviewed. Within this category, environmental pollutants (and particularly air pollutants) were identified as a potentially important risk factor for asthma. Studies concerning associations between dwelling features and occupancy features and asthma reported inconsistent results, highlighting the need for greater research in these areas. Interpreting housing-related asthma triggers through this framework paves the way for the identification and targeting of typologies of housing that might adversely affect asthma, thus addressing multiple characteristics in tandem rather than as isolated elements.

**Supplementary Information:**

The online version contains supplementary material available at 10.1186/s12889-023-16594-8.

## Introduction

Asthma poses a significant health burden globally, with an estimated 300 million people affected by the condition and rates continuing to climb [1]. In 2019 alone, asthma resulted in 455,000 deaths [2] and it is currently the leading chronic disease among children [3]. However, developing strategies to reduce the health burden attributable to asthma is complicated, in part due to the significant heterogeneity observed in its triggers [4].

The link between asthma and living conditions has been well-established [5, 6], with evidence that triggers in home environments, such as dust mites and exposure to fumes, increase asthma risk [3, 7, 8]. Existing reviews, for example by Vernon et al. [9], imply that the most common asthma triggers across countries and continents include allergens in the indoor environment such as mould and pets. Allergens found in the surrounding outdoor environment have also been highlighted as primary asthma triggers [10]. These include air pollution [11], and more specifically traffic-related pollution [12]. Evidence therefore suggests that both the home, and the neighbourhood it is situated in, are important determinants of asthma that should be considered in tandem.

Whilst research identifying triggers has provided crucial insight into the mechanisms through which housing impacts asthma, many triggers identified in this literature represent *symptoms* of housing problems, pertaining to wider or more structural issues in the home or home environment. For example, the growth of mould may be related to inadequate indoor ventilation, building materials or heating and cooling system types. The characteristics of housing that *cause* these symptoms have not yet been comprehensively reviewed, and there are gaps in understanding what these characteristics are. Thus, there is a paucity of housing-focused interventions to reduce asthma and asthma symptoms. Since it is well established that asthma causes are complex and take multiple forms [9], it is possible that health-harming housing characteristics are clustered, interact and overlap, collectively heightening the risk of asthma or asthma symptoms. Identifying these characteristics and mapping the relationships between them would allow for a more effective, long term means of targeting the drivers of asthma in the home simultaneously, rather than addressing their symptoms as isolated elements.

Addressing this, we conceptualise health-harming housing characteristics, and their relationship to one another, through the lens of housing typologies. This theoretical framing is commonly used in architectural studies to distinguish buildings based on relevant categories [13], noting the distinction between building characteristics and architectural styles [14]. For the purposes of this research, a typologies lens is applied to categorise features and characteristics of a house. This includes how it is designed, built, occupied, and where it is located, thus identifying a set of housing characteristics that, in tandem, may contribute to asthma and asthma symptoms. Whilst the role and nature of typologies have been subject to debate [15], this framing helps map existing knowledge, enables the organisation of knowledge into clear categories, and illuminates relationships between these categories [16]. By adopting this framework, key actors concerned with addressing the asthma burden may be better supported to consider how multi-level elements of a home can be layered and interact with each other to potentially increase risk of asthma and asthma symptoms. This knowledge may help clinicians and building providers identify typologies of housing that are problematic for asthma, and anticipate which populations might be particularly vulnerable.

The purpose of this review is therefore twofold: first, it seeks to ascertain what is known about characteristics of health-harming housing in relation to asthma. In doing so, it aims to illuminate areas where more research is needed. Second, it develops a framing for understanding how housing characteristics act together to constitute potentially health-harming housing typologies. Identifying typologies of health-harming housing shifts the focus away from an individual’s circumstances and behaviours and towards more structural issues in the home and home environment that can be more efficiently addressed by public health interventions. This type of framing, aligned with a social determinants of health perspective, is essential to address the burden of asthma attributed to inadequate housing, and the unequal distribution of asthma in the population [17, 18].

## Methods

### Identifying the research question and relevant studies

We followed Arksey and O’Malley’s [19] five stage approach to conducting a scoping review. This has been used extensively in health research. In the first instance, we identified the research question, asking *what is known in the published literature about housing typologies and their contribution to the onset and/or exacerbation of asthma?* We then identified relevant studies using a search strategy whereby search terms were chosen to capture the core research concepts related to housing typologies and asthma (Additional file 1: Appendix A). Alongside ‘asthma’, our search strategy necessitated one of the following in the title: ‘housing’, ‘house’, ‘dwelling’, ‘residence’, or ‘residential’. These terms were used to search three online databases identified as the most relevant to public health and social science research: PubMed, Scopus, and Web of Science. Searches were carried out in August 2022 on studies published between 2012 and 2022. We select a 10-year timeframe to reflect current work in the field and identify gaps where contemporary research is needed. While there are inevitably other housing related factors impacting asthma which will not be retuned in these terms, undertaking a scoping review requires defining clear boundaries to facilitate a comprehensive search of the literature.

### Study selection

**Fig. 1:**
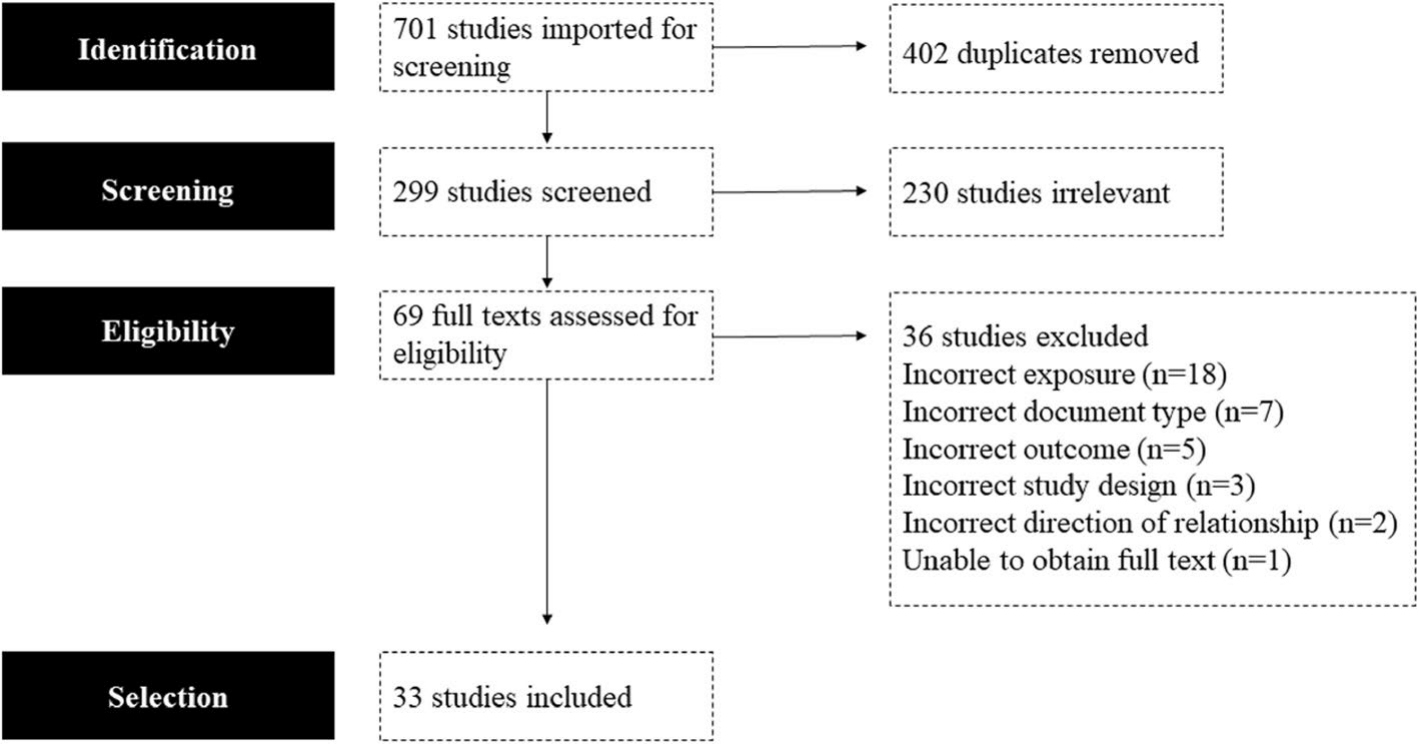
*PRISMA *flow diagram

We selected studies through establishing an inclusion and exclusion criteria based on exposure, outcome, timeframe, population, study design and document type (Additional file 1: Appendix B). The program *Covidence* was used to compile the documents, remove duplicates, and carry out screening. Two reviewers independently performed title and abstract screening to remove results incompatible with the inclusion criteria. If the title and abstract contained insufficient information to assert its compatibility, the full text was obtained before arriving at a decision. One reviewer then carried out full text screening. The second reviewer screened 10 full texts (selected at random) to ensure consistency. Where uncertainty or disagreement about the eligibility of an article arose in each round of screening, the research team was consulted, and a collective decision was reached. The list of studies identified for full text review was assessed and approved by the research team. 701 references were obtained across the three databases (Fig. 1). After excluding 402 duplicates, 299 articles were selected for title and abstract screening, 69 articles were selected for full-text screening, of which 36 were then excluded. 33 articles were selected for inclusion in this review.

### Charting the data and collating, summarising, and reporting the results

Relevant information from the texts being reviewed was extracted into excel, as per the data charting form developed in our protocol (Additional file 1: Appendix C). The 33 studies selected for review were then collated and summarised. For this, a narrative synthesis of results was undertaken to present the findings of this review. Our approach was guided by Braun and Clarke’s [20] method based on six steps, namely familiarisation with data, initial coding, preliminary theme detection, refining themes, defining final themes, and contextualisation of themes. Through this inductive approach, we categorised the characteristics of housing linked to the onset or exacerbation of asthma included in our review. We then established a three-level framework through which to conceptualise how these characteristics are layered.

## Results

The characteristics of the 33 studies reviewed are summarised in Table 1.

**Table 1:** Characteristics of studies selected for inclusion

Authors	Study setting	Sample size	Demographic	Study design
Abebe et al., 2021 [21]	Ethiopia	483	Adults	Case control
Alasauskas et al., 2020 [22]	Lithuania	51,235	Children aged 7 – 17 years	Cross-sectional
Apichainan et al., 2022 [23]	Thailand	658	Children aged 6 – 10 years	Cross-sectional
Brown et al., 2012 [24]	US	224	Children aged 6 – 17 years	Cross-sectional
Bukalasa et al., 2018 [25]	The Netherlands	1,473	Children aged 14 years	Cross-sectional
Delfino et al., 2014 [26]	US	11,390	Children aged 0 – 18 years	Case-crossover
Hartley et al., 2022 [27]	US	617	Children aged 0 – 7 years	Prospective cohort
Hsieh et al., 2019 [28]	Taiwan	7,040	Children age under 18 years	Case control
Hughes et al., 2017 [29]	US	33,201	Children aged 6 – 17 years	Cross-sectional
Hwang et al., 2012 [30]	Korea	1,819	Children aged 6 – 13 years	Cross-sectional
Keet et al., 2015 [31]	US	23,065	Children aged 5—19 years	Cross-sectional
Keet et al., 2017 [32]	US	16,860,716	Children aged 6 – 17 years	Cross-sectional
Kim et al., 2022 [33]	US	16,167	Adults and children aged 1—13 years	Cross-sectional
Knibbs et al., 2018 [7]	Australia	4,144,024	Children up to 14 years	Comparative risk assessment
Kutzora et al., 2019 [34]^a^	Germany		Children aged 5 – 7 years	Time series
Lai et al., 2018 [35]	Australia	88	Adults	Cross-sectional
Liu et al., 2020 [36]	China	9,597	Children aged 3 – 6 years	Cross-sectional
Mazenq et al., 2017 [37]	France	6,250	Adults and children aged 3 years +	Case–control
McDaniel et al., 2020 [38]	US	4,522	Children up to 18 years	Cross-sectional
Mehta et al., 2018 [39]	US	9,554	Adults	Cross-sectional
Mendy et al., 2019 [40]	US	6,488	Adults and children	Cross-sectional
Middleton et al., 2014 [41]	Greece	5,817	Children aged 15 – 17 years	Cross-sectional
Norback et al., 2014 [42]	Sweden	7,554	Adults	Cross-sectional
Nriagu et al., 2012 [43]	US	1,206	Children up to 12 years	Cross-sectional
Oshikata et al., 2021 [44]	Japan	337	Adults and children aged 15 years+	Cohort
Schmidt et al., 2014 [45]	US	2,829	Children aged 2 – 19 years	Randomised controlled trial
Sudan et al., 2017 [46]	Denmark	92,676	Children from gestation—7 years	Cohort
Sun & Sundell, 2013 [47]	US	2,819	Children aged 1 – 8 years	Cross-sectional
Svendsen et al., 2018 [48]	US	5,210	Children aged 9 – 11 years	Cross-sectional
Tieskens et al., 2021 [49]	US	10,000	Children aged 5 years	Simulation
Wang et al., 2013 [50]	China	4,618	Children aged 2 – 7	Cross-sectional
Wang et al., 2017 [51]	Sweden	1,160	Adults	Cross-sectional
Wang et al., 2019 [52]	China	40,279	Children	Cross-sectional

^a^In one instance sample size was unable to be discerned. Attempt was made to contact the corresponding author but this was unsuccessful

Within the 33 studies selected for review, nine housing characteristics were identified: residential green space; environmental pollutants; urban density; heating, ventilation, and air conditioning (HVAC); building type and materials; housing age and state of repair; tenure; crowding; and appliances. These characteristics correspond to three levels of a home, that is, its surrounding environment (locational features), the house itself (dwelling features), and the internal conditions of the home (occupancy features). Associations between the housing characteristics and asthma as reviewed in this section are summarised in Table 2.

**Table 2:** Summary of associations between housing characteristics and asthma

	**Locational features**			**Dwelling features**			**Occupancy features**		
	***Residential green space***	***Environmental pollutants***	***Urban density***	***HVAC***^***a***^	***Building type and materials***	***Housing age and state of repair***	***Tenure***	***Crowding***	***Appliances***
***Asthma among children***									
Alasauskas et al*.,* 2020 [22]	✓	✓							
Apichainan et al*.,* 2022 [23]		✓			X	X	X		X
Brown et al*.,* 2012 [24]		✓							
Bukalasa et al*.,* 2018 [25]		X							
Delfino et al*.,* 2014 [26]		✓							
Hartley et al*.,* 2022 [27]	✓								
Hsieh et al*.,* 2019 [28]	✓								
Hughes et al*.,* 2017 [29]						✓	✓	X	
Hwang et al*.,* 2012 [30]		***○***	✓				✓		
Keet et al*.,* 2015 [31]			✓						
Keet et al*.,* 2017 [32]			X						
Knibbs et al*.,* 2018 [7]									✓
Kutzora et al*.,* 2019 [34]								X	
Liu et al*.,* 2020 [36]		✓							
McDaniel et al*.,* 2020 [38]			✓						
Middleton et al*.,* 2014 [41]		✓							
Nriagu et al*.,* 2012 [43]				***○***		✓			✓
Schmidt et al*.,* 2014 [45]							✓		
Sudan et al*.,* 2017 [46]		X							
Sun & Sundell, 2013 [47]				✓	***○***	X			
Svendsen et al*.,* 2018 [48]		✓		***○***					✓
Tieskens et al*.,* 2021 [49]				***○***					✓
Wang et al*.,* 2013 [50]		X	X	X	X		X		X
Wang et al*.,* 2019 [51]			✓			✓	X		
***Asthma among adults***									
Abebe et al*.,* 2021 [21]				✓	X			X	✓
Lai et al*.,* 2018 [35]		***○***							
Mehta et al*.,* 2018 [39]							✓		
Norback et al*.,* 2014 [42]				X		X	X	X	
Wang et al*.,* 2017 [52]				✓	✓	X			
***Asthma among children and adults***									
Kim et al*.,* 2022 [33]							***○***		
Mazenq et al*.,* 2017 [37]		✓							
Mendy et al*.,* 2019 [40]		✓							
Oshikata et al*.,* 2021 [44]							✓		

✓ = Association found

X = No association found

**○** = Both association and no association found

^a^Heating, ventilation and air-conditioning systems are defined as "the equipment, distribution systems, and terminals that provide, either collectively or individually, the processes of heating, ventilating, or air conditioning to a building or portion of a building" [53]

### Locational features

#### Environmental pollutants

Of the research on asthma and environmental pollutants reviewed in this study, proximity of the home to air pollutants (mostly nitrogen dioxide and pollutants associated with traffic) were the main area of focus. Other indicators of the presence of air pollutants were proximity to factories [23], harmful facilities [30], fuel oil powerplants [41], industry/business [50] and magnetic fields [46]. Studies analysing soil or land pollutants in relation to asthma concerned pesticide exposures [25, 48].

Only one study on environmental pollutants focused on adults. Lai et al*.* [35] found that nitrogen dioxide exposure in the surrounding environment was positively associated with more asthma-related emergency healthcare visits, but with no significant association between distance from major road (taken as a proxy for air pollution) and healthcare usage. A small sample size of 88 participants may limit validity. Two larger studies concerning children and adults support Lai et al*.*’s [35] conclusions [37, 40]. The authors find a positive association between exposure to nitrogen dioxide and particulate matter and admissions to emergency, although Mazenq's [37] findings of particulate matter were only observed for children.

Eleven additional studies explored associations between environmental pollutants and childhood asthma. Again, air pollutants related to traffic were prevalent (e.g., [26]). Seven studies in this area reported significant associations [22–24, 26, 36, 41, 48], three reported no significant associations [25, 46, 50], and one reported both evidence of significant and non-significant associations [30]. Differences in the measurement of exposure, and the potentially moderating effects of other housing conditions might explain some inconsistency in findings. For example, Lui et al*.* [36] found living on lower floors and having less ventilation increased the strength of positive associations between environmental pollutants and asthma. Amongst the studies reporting significant associations, there was generally evidence of positive relationships whereby increased exposure to pollutants (specifically those associated with proximity to traffic) was found to be related to asthma or asthma symptoms. Overall, results imply that environmental pollutants in the surrounding environment – particularly air pollutants related to traffic – play an important role in asthma and asthma symptoms.

#### Residential green space

Access to trees, parks and gardens where people live has been related to respiratory health [54]. On one hand, green spaces are associated with better air quality, and provide opportunities for exercise and/or restorative activities [55]. On the other hand, green spaces can be a source of pollen and allergy that trigger the symptoms of people with asthma [55]. While all three studies concerning the association between residential green space and childhood asthma in this review reported a significant association, the direction of the relationship was mixed, reflecting the complexity of this relationship. One study found that asthma risk decreased with proximity to greenspace, which the authors attributed to lower air pollutants [22]. In this study, greenspace was measured as the distance to areas with trees and bushes. On the contrary, Hsieh et al*.* [28] noted an increase of risk of asthma amongst those living near residential greenness, measured through the Normalized Difference Vegetation Index (NDVI). However, the authors found no significant difference in risk of asthma based on *how* close the residence was (i.e., within 200, 400 and 800 meters from the home). Hartley et al*.* [27], also quantifying residential greenspace using the NDVI, similarly found the likelihood of developing asthma increased with proximity to greenness. It should be noted that the sample was children who were sensitised to common allergens.

#### Urban density

Whilst definitions of urban density vary, it broadly indicates the number of people (and, by proxy, the amount of infrastructure and other buildings and facilities) within a given area [56]. Six studies explored associations between urban density and childhood asthma, with mixed results. Four studies found significant associations [30, 31, 38, 52], but again with contrasting evidence surrounding the direction of associations. Hwang et al*.* [30] and McDaniel et al*.* [38] found living outside of cities heightened the risk of asthma. Keet et al*.* [31] found a positive association between residing in poor urban areas and asthma-related emergency visits and hospitalisations, but this did not hold for asthma *prevalence*. A later study by the same authors found demographic composition of residents accounted for the positive association observed between some inner city areas and asthma prevalence [32]. Differing interpretations of urban density and its context (i.e., urban, suburban or rural) across studies might account for the inconsistent results.

### Dwelling features

#### Housing age and state of repair

Six studies analysed associations between housing age and asthma, with mixed results. Of studies concerning childhood asthma, there was evidence of both significant [43, 52] and non-significant [23, 47] associations. Of those reporting significant findings, a positive relationship was noted whereby older housing increased asthma prevalence. On the contrary, studies concerning housing age and asthma amongst adults did not report a significant association [42, 51]. It should be questioned how far associations are indebted to housing conditions rather than age itself. Indeed, Hughes et al*.* [29] found a positive association between poor housing quality and childhood asthma.

#### Building type and materials

Of the four studies concerning building type (e.g., apartment or house) and asthma, living in a trailer was the only dwelling type to be found to be associated with asthma risk. One to eight year old children in this building type determined to be at 50% higher risk of asthma than those in other dwellings [47]. Characteristics of trailers might explain this finding: compared to other dwellings like single family homes, trailers were more likely to have natural ventilation systems, dampness problems, and differences in foundations, walls and flooring [47]. Two studies analysed associations between building materials (e.g., brick, wood) and childhood asthma. Wang et al*.* [50] found no evidence of an association between building foundations, brick facades and asthma, and Wang et al*.* [52] did not report any statistically significant associations between floor materials, wood wall materials and asthma. Significant positive associations were found between living in a dwelling with crawlspace (basement or underground room) and asthma symptoms [51].

### Occupancy features

#### Tenure

Tenure refers to the arrangement by which housing is obtained, for example owned or rented from private parties or the state [57]. Two studies analysed associations between tenure and asthma amongst adults. Mehta et al*.* [39] reported positive associations between living in public housing and asthma, and receiving rental assistance and asthma. Norback et al*.* [42] reported no association between tenure and asthma when considering private and public rental housing and homeownership.

Two further studies analysed associations between tenure and asthma among both adults and children. Kim et al*.* [33] found that, compared to homeownership, adults residing in public housing were at higher risk of asthma and asthma symptoms, but this did not hold amongst those receiving rental assistance. Amongst children, associations with asthma and tenure were non-significant when considering both residents of public housing, and rental-assistance recipients [33]. Oshikata et al*.* [44] study of children and adults who had resided in temporary housing for at least a year reported that prevalence of asthma among residents and past-residents exceeded twice the national average, with new cases diagnosed after residents moved out. The authors attributed this to environmental features which led to the development of asthma either whilst residing in temporary housing or in the period that followed.

Six further studies analysed associations between tenure and childhood asthma. There was consensus between Hwang et al*.* [30] and Hughes et al*.* [29] that homeownership was associated with a lower prevalence of asthma compared to renting, but Apichainan et al*.* [23], Wang et al. [50] and Wang et al*.* [52] found no significant difference in asthma between owners and renters. Comparing differences between renters, Schmidt et al*.*’s [45] randomised control trial involving low-income public-housing residents moving to subsidised private rental housing indicated a significant negative association between relocation and asthma. This was contrary to expectations that this shift would improve health outcomes. These studies emphasise that relationships between asthma and tenure are not straightforward.

#### Crowding

Crowding refers to the availability of rooms and space relative to inhabitants. Measures of overcrowding can be objective (i.e., derived from household size, number of rooms or bedrooms, and the ages, sexes and relationships of occupants) or subjective (i.e., based on self reported lack of sufficient space relative to household members or their needs) [58]. The former were applied in the four studies reviewed in this paper: Norback et al. [42], Abebe et al. [21], Hughes et al. [29] and Kutzora et al. [34] consider the number of persons per room or bedroom, whilst the latter also considered floor space (with less than 20 square metres per person indicative of crowding). None of the studies concerning associations between household crowding and asthma among either children [29, 34] or adults [21, 42] reported significant associations. One reported an association between crowding and cough, a common asthma symptom [34].

#### Heating ventilation and air conditioning

Heating, ventilation and air-conditioning systems are defined as "the equipment, distribution systems, and terminals that provide, either collectively or individually, the processes of heating, ventilating, or air conditioning to a building or portion of a building" [53]. The literature reviewed includes variation in system type (e.g. central or space conditioning) and fuel source (e.g. fireplace, electricity or gas), which may contribute to the mixed results noted. Of the studies on associations between asthma and heating source, Svendsen et al*.* [48] found that fireplace heat was both positively and negatively associated with childhood asthma, depending on the measure (e.g., prevalence, or severity or symptoms). Nriagu et al*.* [43] did not report significant associations between kerosene space heaters, wall heaters and gas fireplaces and childhood asthma, although they noted that there was a limited number of households using these sources. Norback et al*.* [42] reported no significant association between energy type used for space heating and asthma amongst adults. Wang et al*.*’s [50] study of children also reported no significant associations between heating type and asthma, though categories were restricted to 'central heating' and ‘other’.

There was consensus between Wang et al*.* [51] and Abebe et al*.* [21] that low ventilation flow was a risk factor for adult asthma. On the contrary, Norback et al*.* [42] found no significant association between type of ventilation and airing habits and asthma. Nriagu et al*.* [43] found that reports of missing or inoperable windows were not significantly associated with childhood asthma.

Studies of children found positive associations between central air conditioning and asthma [47, 48], with the study by Svendsen et al. [48] also reporting a positive association between humidifier use and asthma prevalence. Nriagu et al*.* [43] reported that whilst presence of a working air conditioner was a not a protective factor against asthma, exhaust fans were negatively correlated. This was reitterated by Tieskens et al*.* [49] who predicted that intense retrofitting (with kitchen and bathroom exhaust fans and a mechanical ventilation system) was negatively correlated with serious childhood asthma events per year.

#### Appliances

Appliances in this review are classified as electrical or fuel using equipment that can negatively impact indoor air quality, which are not for the purpose of modifying the temperature, fresh air or humidity within the house. Examples include cooking, washing and drying electrical equipment. However, only studies concerning cooking appliances were returned by the search strategy.

Abebe et al*.* [21] found that wood and agricultural residues for cooking were positively correlated with asthma amongst adults, whilst six further studies considering associations between cooking sources and childhood asthma indicated mixed results. No association was found by Apichainan et al*.* [23] analysis of asthma and charcoal stoves, Wang et al*.* [52] analysis of asthma and gas/ electricity, or Wang et al*.* [50] analysis of asthma and use of gas, electricity, coal or wood for cooking. Nriagu et al*.* [43] found no associations between using wood burning stoves and asthma but reported a significant negative association between using a gas stove and asthma. The latter was reiterated by Knibbs et al*.* [7]. Svendsen et al*.* [48] reported mixed evidence for this association, finding a positive correlation between cooking with gas stoves and asthma prevalence, but reported a negative association with asthma severity. Further, the authors found that woodstove use was associated with increased severity of asthma symptoms, reiterating the findings of Abebe et al*.* [21] concerning adults. Findings therefore indicate cooking fuel source – particularly gas – might play a role in exhasberating asthma and asthma symptoms.

## Discussion

This study has scoped the existing literature on housing typologies and asthma. Where housing has been widely recognised as an important determinant of asthma [59], this review of 33 studies demonstrates the diversity and complexity of mechanisms behind this association.

Of the three levels of the home put forward in our housing typologies framework, the association between asthma and locational features was evidenced most clearly. Within this category, associations between environmental pollutants (mostly concerning air pollutants) and asthma have received the greatest scholarly attention, with 11 of 14 studies in our review finding a significant association between pollutants and asthma or asthma symptoms. An additional four of six studies reported an association between urban density and asthma, although the direction of these associations was mixed. This reflects the complexity of the pathways between urban density and health [60]. Three studies analysed asthma in relation to residential greenspace, and while all identified significant associations, these were both positive and negative. This is consistent with literature and with others reviews in this area, which have highlighted mechanisms associated with greenspace that might improve (for example, through improved air quality), or worsen (for example, through exposure to pollen and allergens), asthma symptoms [55]. The findings indicate the potentially important role of locational features in asthma outcomes, whilst also noting that exposures were highly varied (e.g., from proximity to power plants to exposure to pesticides). Unpacking specific mechanisms behind the associations requires more evidence across each of these domains. Nevertheless, findings suggest that the location of a home likely goes some way in explaining the uneven distribution of asthma within in a population: evidence suggests that low-income populations, minority groups and persons in social housing are often housed in places with e.g., limited greenspace, higher building density, and poorer outdoor air quality [61, 62].

Evidence for associations between dwelling features and asthma was less clear. Of the five studies analysing associations between building types and materials and asthma, living in a trailer was the only building type found to have a significant positive association with childhood asthma [47]. Features of trailers compared to other housing types, such as more dampness, might explain this association. Further, no associations were found between other dwelling types studied (such as living in an apartment or house) and building materials and asthma. Results were mixed for the seven studies analysing associations between asthma and dwelling age or state of repair and the eight studies analysing associations between HVAC and asthma. This might point to complexity of exposures, for example older housing may be of a better build quality in some places than others depending on building standards and materials used in the respective era. Other unmeasurable factors, such as climatic variability across the study contexts, might also go some way in complicating findings.

Studies analysing the relationship between occupancy features and asthma also indicated mixed findings. No significant associations were found in the four studies analysing associations between asthma and crowding, whereas six of the 10 studies analysing associations between tenure and asthma indicated significant findings. Homeownership was typically emphasised as the ‘healthiest’ tenure in our findings, although associations between private and public rental housing were mixed. The one study researching temporary housing highlighted this as a key risk factor for asthma both during and after exposure [44]. Allergen exposure and sensitisation are possible explanations. Associations between appliances and asthma – particularly gas cookers – were also highlighted as a potentially important risk factor, underscoring the relative disadvantage experienced by groups facing barriers to upgrading appliances due to income constraints and/or rental status, amongst other factors [63].

**Fig. 2:**
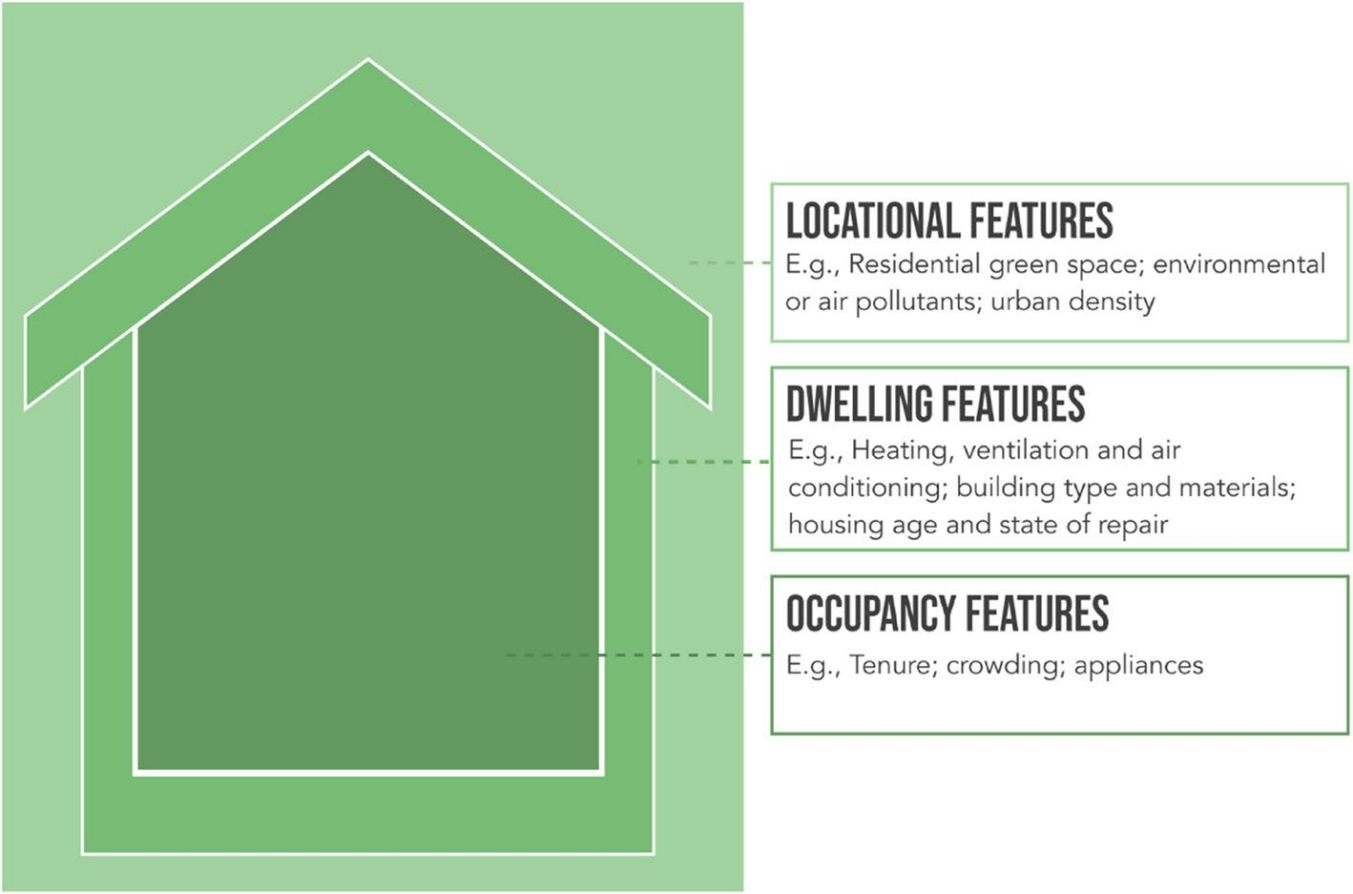
Housing typologies framework. *Note.* This figure maps the housing characteristics covered in this review (noting that this is not an exhaustive list of the housing conditions that may contribute to asthma risk) and illustrates how these can exist on multiple levels to potentially enhance asthma risk and/or exacerbate asthma symptoms

Drawing on the three levels of a home identified in this study (namely, locational, dwelling and occupancy features), we established a housing typologies framework that illustrates how health-harming housing characteristics can be clustered and layered (Fig. 2), cumulatively increasing risk of asthma onset or exacerbation. This framework paves the way for future research to identify *typologies* of health-harming housing, addressing multiple housing-related asthma triggers in tandem, rather than as isolated elements. Identifying typologies of housing which increase asthma risk is important given the heterogeneity of asthma triggers [4].

Identification of typologies of housing that may be particularly harmful in relation to asthma offers the opportunity for more effective remediation of multiple risk factors simultaneously. Whilst this review has highlighted the need for further research across all housing characteristics discussed, provisional findings from the studies synthesised imply that a home located in proximity to traffic, with inadequate ventilation, that is not privately owned and that uses gas stoves for cooking, for example, is a housing typology that might exacerbate asthma or asthma symptoms. Characteristics defined in this framework are not an exhaustive list but a starting point, reflecting nine key characteristics of the home that have been analysed in association to asthma in the 33 studies identified in this review.

These findings are notwithstanding limitations. Across all housing characteristics studied, there are notable differences in measurement of both exposure and outcomes. For example, among studies concerning traffic-related pollutants, measures typically applied crude thresholds to indicate proximity to major roads, traffic or traffic-facilities. These varied from 500 [26], 200 [50] and 100 [36] meters of the home. Other measurements relied on self-report, for example “little” or “much” traffic [30]. More consistency in measurements might reduce contradictions in findings and enable a clearer identification of asthma risks and triggers.

While our review has covered a range of recent literature on associations between housing and asthma, it would be remiss to not acknowledge work undertaken that, due to the limited scope of our search strategy, was not included in the papers selected for inclusion. One example is a body of research undertaken in New Zealand on relationships between asthma and the level of neighbourhood vegetation [64], improved home heating [65, 66] home insulation [67] and housing quality [68]. Insights from this body of literature would no doubt further illuminate relationships between housing asthma. In addition, while it was beyond the scope of this review to consider the role of financial and socio-demographic characteristics, and psycho-social stresses, in shaping the associations between housing typologies and asthma, it is important to note that these factors might play a crucial role in modifying risk [31, 69].

## Conclusion

This scoping review sought to compile recent research on what is known about housing typologies and their contribution to the onset and/or exacerbation of asthma. Expanding upon the established literature on housing and asthma triggers, which largely focuses on *symptoms* of health-harming housing, such as pests and mould, this review has instead concentrated on the housing characteristics which may *cause* these triggers.

Sorting these characteristics into a conceptual framework of housing typologies paves the way for future research to examine housing characteristics which might layer to cumulatively increase risk of asthma or asthma symptoms. This provisional framework also has several potential applications. It could underpin a checklist for use by building practitioners and housing providers that aligns building standards with evidence on health effects, or be applied to develop resources that aid in the identification of asthma triggers in the home for use in clinical settings. The framework could also assist with developing criteria to help households identify asthma triggers in their homes. Possible benefits are enabling more effective home remediations, and informed decisions when searching for new residences. Finally, in line with a social determinants of health framing, this review focuses on the distal causes of asthma risk. Identifying and targeting these typologies and addressing housing risk factors *in tandem* offers an effective way to improve public health, reduce population-level asthma disparities, and address inequalities.

### Supplementary Information

**Additional file 1: Appendix A.** Search strings by database. **Appendix B.** Inclusion criteria. **Appendix C.** Data extraction template.

## Data Availability

The datasets analysed during the current study available from the corresponding author on reasonable request.

## References

[1] Dharmage SC, Perret JL, Custovic A (2019). Epidemiology of Asthma in Children and Adults. Front Pediatr..

[2] World Health Organisation. Asthma. 2022. Available from: https://www.who.int/news-room/fact-sheets/detail/asthma. Accessed 1 May 2023.

[3] Krieger J (2010). Home is Where the Triggers Are: Increasing Asthma Control by Improving the Home Environment. Pediatr Allergy Immunol Pulmonol..

[4] Frey U, Suki B (2008). Complexity of chronic asthma and chronic obstructive pulmonary disease: implications for risk assessment, and disease progression and control. Lancet..

[5] Calderón MA, Linneberg A, Kleine-Tebbe J, De Blay F, Hernandez Fernandez de Rojas D, Virchow JC (2015). Respiratory allergy caused by house dust mites: What do we really know?. J Allergy Clin Immunol..

[6] Quansah R, Jaakkola MS, Hugg TT, Heikkinen SAM, Jaakkola JJ (2012). Residential dampness and molds and the risk of developing asthma: a systematic review and meta-analysis. PLoS ONE.

[7] Knibbs LD, Woldeyohannes S, Marks GB, Cowie CT (2018). Damp housing, gas stoves, and the burden of childhood asthma in Australia. Med J Aust.

[8] Krieger J, Jacobs DE, Ashley PJ, Baeder A, Chew GL, Dearborn D (2010). Housing Interventions and Control of Asthma-Related Indoor Biologic Agents: A Review of the Evidence. J Public Health Manage Pract..

[9] Vernon MK, Wiklund I, Bell JA, Dale P, Chapman KR (2012). What Do We Know about Asthma Triggers? A Review of the Literature. J Asthma..

[10] Gautier C, Charpin D. Environmental triggers and avoidance in the management of asthma. J Asthma Allergy. 2017;10:47–56. 10.2147/JAA.S121276 PMC5349698 28331347

[11] Tzivian L (2011). Outdoor Air Pollution and Asthma in Children. J Asthma..

[12] Pollock J, Shi L, Gimbel RW (2017). Outdoor Environment and Pediatric Asthma: An Update on the Evidence from North America. Can Respir J..

[13] Gulgonen A, Laisney F (1982). Contextual Approaches to Typology at the Ecole des Beaux-Arts. J Archit Educ..

[14] Remali AM, Salama AM, Wiedmann F, Ibrahim HG (2016). A chronological exploration of the evolution of housing typologies in Gulf cities. City Territ Archit..

[15] Bandini M (1984). Typology as a form of convention. AA Files..

[16] Tiryakian E (1968). Typologies. Int Encyclopedia Soc Sci..

[17] Canino G, McQuaid EL, Rand CS (2009). Addressing asthma health disparities: A multilevel challenge. J Allergy Clin Immunol..

[18] Curtis LM, Wolf MS, Weiss KB, Grammer LC (2012). The Impact of Health Literacy and Socioeconomic Status on Asthma Disparities. J Asthma..

[19] Arksey H, O’Malley L (2005). Scoping studies: towards a methodological framework. International J Soc Res Method..

[20] Braun V, Clarke V (2006). Using thematic analysis in psychology. Qual Res Psychol..

[21] Abebe Y, Ali A, Kumie A, Haile T, Tamire M, Addissie A (2021). Determinants of asthma in Ethiopia: age and sex matched case control study with special reference to household fuel exposure and housing characteristics. Asthma Res Pract.

[22] Alasauskas S, Ustinaviciene R, Kavaliauskas M. Residential Links to Air Pollution and School Children with Asthma in Vilnius (Population Study). Medicina (Kaunas). 2020;56(7):1–11. 10.3390/medicina56070346 PMC7404686 32668717

[23] Apichainan N, Norkaew S, Taneepanichskul N (2022). Residential environment in relation to self-report of respiratory and asthma symptoms among primary school children in a high-polluted urban area. Sci Rep.

[24] Brown MS, Sarnat SE, DeMuth KA, Brown LAS, Whitlock DR, Brown SW (2012). Residential proximity to a major roadway is associated with features of asthma control in children. PLoS ONE.

[25] Bukalasa JS, Brunekreef B, Brouwer M, Koppelman GH, Wijga AH, Huss A (2018). Associations of residential exposure to agricultural pesticides with asthma prevalence in adolescence: The PIAMA birth cohort. Environ Int.

[26] Delfino RJ, Wu J, Tjoa T, Gullesserian SK, Nickerson B, Gillen DL (2014). Asthma morbidity and ambient air pollution: effect modification by residential traffic-related air pollution. Epidemiology.

[27] Hartley K, Ryan PH, Gillespie GL, Perazzo J, Wright JM, Rice GE (2022). Residential greenness, asthma, and lung function among children at high risk of allergic sensitization: a prospective cohort study. Environ Health.

[28] Hsieh CJ, Yu PY, Tai CJ, Jan RH, Wen TH, Lin SW, et al. Association between the First Occurrence of Asthma and Residential Greenness in Children and Teenagers in Taiwan. Int J Environ Res Public Health. 2019;16(12):1–11. 10.3390/ijerph16122076 PMC6616887 31212779

[29] Hughes HK, Matsui EC, Tschudy MM, Pollack CE, Keet CA (2017). Pediatric Asthma Health Disparities: Race, Hardship, Housing, and Asthma in a National Survey. Acad Pediatr.

[30] Hwang GS, Choi JW, Yoo Y, Choung JT, Yoon CS (2012). Residential environmental risk factors for childhood asthma prevalence in metropolitan and semirural cities in Korea. Asia Pac J Public Health.

[31] Keet CA, McCormack MC, Pollack CE, Peng RD, McGowan E, Matsui EC (2015). Neighborhood poverty, urban residence, race/ethnicity, and asthma: Rethinking the inner-city asthma epidemic. J Allergy Clin Immunol.

[32] Keet CA, Matsui EC, McCormack MC, Peng RD (2017). Urban residence, neighborhood poverty, race/ethnicity, and asthma morbidity among children on Medicaid. J Allergy Clin Immunol.

[33] Kim B, Mulready-Ward C, Thorpe LE, Titus AR (2022). Housing environments and asthma outcomes within population-based samples of adults and children in NYC. Prev Med.

[34] Kutzora S, Puerto Valencia L, Weber A, Huß J, Hendrowarsito L, Nennstiel-Ratzel U (2019). Residential crowding and asthma in preschool children, a cross-sectional study. Allergol Immunopathol (Madr).

[35] Lai VWY, Bowatte G, Knibbs LD, Rangamuwa K, Young A, Dharmage S (2018). Residential NO(2) exposure is associated with urgent healthcare use in a thunderstorm asthma cohort. Asia Pac Allergy.

[36] Liu W, Cai J, Huang C, Chang J (2020). Residence proximity to traffic-related facilities is associated with childhood asthma and rhinitis in Shandong. China Environ Int.

[37] Mazenq J, Dubus JC, Gaudart J, Charpin D, Viudes G, Noel G (2017). City housing atmospheric pollutant impact on emergency visit for asthma: A classification and regression tree approach. Respir Med.

[38] McDaniel JT, McDermott RJ, Martinasek MP, White RM (2020). Prevalence of childhood asthma in US military and non-military families: Comparisons by rural-urban residence and geographic region. Chronic Illn.

[39] Mehta AJ, Dooley DP, Kane J, Reid M, Shah SN (2018). Subsidized Housing and Adult Asthma in Boston, 2010–2015. Am J Public Health.

[40] Mendy A, Wilkerson J, Salo PM, Weir CH, Feinstein L, Zeldin DC (2019). Synergistic Association of House Endotoxin Exposure and Ambient Air Pollution with Asthma Outcomes. Am J Respir Crit Care Med.

[41] Middleton N, Kolokotroni O, Lamnisos D, Koutrakis P, Yiallouros PK (2014). Prevalence of asthma and respiratory symptoms in 15–17 year-old Greek-Cypriots by proximity of their community of residence to power plants: Cyprus 2006–07. Public Health.

[42] Norback D, Lampa E, Engvall K. Asthma, Allergy and Eczema among Adults in Multifamily Houses in Stockholm (3-HE Study) - Associations with Building Characteristics, Home Environment and Energy Use for Heating. PLOS ONE. 2014;9(12):1–11. 10.1371/journal.pone.0112960 PMC4257552 25479551

[43] Nriagu J, Martin J, Smith P, Socier D (2012). Residential hazards, high asthma prevalence and multimorbidity among children in Saginaw. Michigan Sci Total Environ.

[44] Oshikata C, Watanabe M, Ishida M, Kobayashi S, Hashimoto K, Kobayashi N (2021). Association between Temporary Housing Habitation after the 2011 Japan Earthquake and Mite Allergen Sensitization and Asthma Development. Int Arch Allergy Immunol.

[45] Schmidt NM, Lincoln AK, Nguyen QC, Acevedo-Garcia D, Osypuk TL (2014). Examining mediators of housing mobility on adolescent asthma: results from a housing voucher experiment. Soc Sci Med.

[46] Sudan M, Arah OA, Becker T, Levy Y, Sigsgaard T, Olsen J (2017). Re-examining the association between residential exposure to magnetic fields from power lines and childhood asthma in the Danish National Birth Cohort. PLoS ONE.

[47] Sun Y, Sundell J (2013). On Associations between Housing Characteristics, Dampness and Asthma and Allergies among Children in Northeast Texas. Indoor Built Environ.

[48] Svendsen ER, Gonzales M, Commodore A (2018). The role of the indoor environment: Residential determinants of allergy, asthma and pulmonary function in children from a US-Mexico border community. Sci Total Environ.

[49] Tieskens KF, Milando CW, Underhill LJ, Vermeer K, Levy JI, Fabian MP (2021). The impact of energy retrofits on pediatric asthma exacerbation in a Boston multi-family housing complex: a systems science approach. Environ Health.

[50] Wang T, Zhao Z, Yao H, Wang S, Norback D, Chen J (2013). Housing characteristics and indoor environment in relation to children’s asthma, allergic diseases and pneumonia in Urumqi. Chin Sci Bull.

[51] Wang J, Engvall K, Smedje G, Nilsson H, Norback D (2017). Current wheeze, asthma, respiratory infections, and rhinitis among adults in relation to inspection data and indoor measurements in single-family houses in SwedenThe BETSI study. Indoor Air.

[52] Wang J, Zhao Z, Zhang Y, Li B, Huang C, Zhang X, et al. Asthma, allergic rhinitis and eczema among parents of preschool children in relation to climate, and dampness and mold in dwellings in China. Environ Int. 2019;130:1–11. 10.1016/j.envint.2019.104910 31226554

[53] American Society of Heating, Refrigerating and Air-Conditioning Engineers. ASHRAE Terminology: A Comprehensive Glossary of Terms for the Built Environment. 2023. Available from: https://terminology.ashrae.org/.

[54] Mueller W, Milner J, Loh M, Vardoulakis S, Wilkinson P (2022). Exposure to urban greenspace and pathways to respiratory health: An exploratory systematic review. Sci Total Environ..

[55] World Health Organization (2016). Urban green spaces and health.

[56] Duranton G, Puga D (2020). The Economics of Urban Density. J Econ Perspect..

[57] Mansour A, Bentley R, Baker E, Li A, Martino E, Clair A (2022). Housing and health: an updated glossary. J Epidemiol Community Health.

[58] Sunega P, Lux M (2016). Subjective perception versus objective indicators of overcrowding and housing affordability. J Housing Built Environ.

[59] Bryant-Stephens TC, Strane D, Robinson EK, Bhambhani S, Kenyon CC (2021). Housing and asthma disparities. J Allergy Clin Immunol..

[60] Carnegie ER, Inglis G, Taylor A, Bak-Klimek A, Okoye O (2022). Is Population Density Associated with Non-Communicable Disease in Western Developed Countries? A Systematic Review. IJERPH..

[61] Braubach M, Fairburn J (2010). Social inequities in environmental risks associated with housing and residential location—a review of evidence. Eur J Pub Health.

[62] Ferguson L, Taylor J, Davies M, Shrubsole C, Symonds P, Dimitroulopoulou S (2020). Exposure to indoor air pollution across socio-economic groups in high-income countries: A scoping review of the literature and a modelling methodology. Environ Int..

[63] Easthope H (2014). Making a Rental Property Home. Housing Stud..

[64] Donovan GH, Gatziolis D, Longley I, Douwes J (2018). Vegetation diversity protects against childhood asthma: results from a large New Zealand birth cohort. Nature Plants..

[65] Free S, Howden-Chapman P, Pierse N, Viggers H, the Housing, Heating and Health Study Research Team (2010). the Housing, More effective home heating reduces school absences for children with asthma. J Epidemiol Commun Health..

[66] Howden-Chapman P, Pierse N, Nicholls S, Gillespie-Bennett J, Viggers H, Cunningham M (2008). Effects of improved home heating on asthma in community dwelling children: randomised controlled trial. BMJ..

[67] Fyfe C, Barnard LT, Douwes J, Howden‐Chapman P, Crane J. Retrofitting home insulation reduces incidence and severity of chronic respiratory disease. Indoor Air. 2022;32(8). Accessed 2023 Mar 20. Available from: https://onlinelibrary.wiley.com/doi/10.1111/ina.13101. 10.1111/ina.13101 PMC9545372 36040274

[68] Keall MD, Crane J, Baker MG, Wickens K, Howden-Chapman P, Cunningham M (2012). A measure for quantifying the impact of housing quality on respiratory health: a cross-sectional study. Environ Health..

[69] Sandel M, Wright RJ (2006). When home is where the stress is: expanding the dimensions of housing that influence asthma morbidity. Arch Dis Childhood..

