# Supplementary material for: Housing typologies and asthma: a scoping review
Source: BMC Public Health. 2023 Sep 11;23:1766. doi: 10.1186/s12889-023-16594-8 (PMC10494403; doi:10.1186/s12889-023-16594-8)
Supplement: Supplementary file 1 — Additional file 1: Appendix A. Search strings by database. Appendix B. Inclusion criteria. Appendix C. Data extraction template. [file 12889_2023_16594_MOESM1_ESM.docx]

**APPENDICES**

**Appendix A: Search strings by database**

*Scopus*

TITLE (housing OR house OR dwelling OR residence OR residential) AND TITLE (asthma) AND PUBYEAR > 2012 AND PUBYEAR < 2022 AND ( LIMIT-TO ( DOCTYPE , "ar" ) )

*Web of Science*

((((((TI=(housing)) OR TI=(house)) OR TI=(dwelling)) OR TI=(residence)) OR TI=(residential)) AND TI=(asthma)) AND PY=(2012-2022)

Manually select *Document Types: Article*

*PubMed*

((((((housing[Title]) OR (house[Title])) OR (dwelling[Title])) OR (residence[Title])) OR (residential[Title])) AND (asthma[Title])) AND (("2012/01/01"[Date - Publication] : "2022/12/31"[Date - Publication]))

Automatically selects articles as the single document type.

**Appendix B: Inclusion criteria**

| **Criteria** | **Description** |
| --- | --- |
| Exposure | A minimum of one identified characteristic of housing examined in relation to asthma. Since an objective of this study was to establish a provisional framework of typologies, our criteria did not provide definitive guidance on which housing characterises were to be included. This was developed and refined through an interactive process. |
| Outcome | Diagnosis of, or symptoms consistent with, asthma. Prescriptions and asthma therapies as indicators of asthma were excluded. Asthma-related hospital admissions and emergency department visits were included. Other respiratory disorders were excluded. |
| Timeframe | Between 2012 and 2022. We limit the search to the last 10 years due to resourcing and constant developments in the field. |
| Population | Human population with no restriction by geographical location or age. Studies where the exposure took place *solely* in the gestation period are not included. |
| Study design | Primary analyses of all study designs. |
| Document type | Peer-reviewed articles published in English, excluding e.g., reviews or letters to the editor. |

**Appendix C: Data extraction template**

| 1 | Study characteristics | Title, author(s), year of publication, setting |
| --- | --- | --- |
| 2 | Study population | Size, demographic characteristics |
| 3 | Study design | Study design, duration of study |
| 4 | Exposure | Categorisation and measurement of exposure |
| 5 | Outcome | Categorisation of outcome, measurement of outcome |
| 6 | Aims | Summary of study aims |
| 7 | Results | Summary of key findings |
